# The Authenticity of Digital News Coverage in the Mainstream Media in Japan

**DOI:** 10.1007/s42979-022-01237-3

**Published:** 2022-06-25

**Authors:** Terumi Miyazoe, Shinichi Sato

**Affiliations:** 1 Tokyo University of Science, Tokyo, Japan; 2 Nihon Fukushi University, Aichi, Japan

**Keywords:** Digital media literacy, Fake news, Misinformation, Mainstream media, Gap analysis, Japan

## Abstract

This research examines the authenticity of digital news coverage in the mainstream media in Japan via a specific case study, namely ‘Doctoral Students Have Decreased by Half’. In research, ‘fake news’ comprises two elements, facticity and intentionality; this study focuses on facticity or misinformation. Studies regarding ‘fake news’ are abundant, but those focussing on the mainstream digital media and Japan are highly scarce. This study applied a gap analysis, a comparison between the expected original information and the actual news coverage in a reversed direction. The study first detected and examined the original governmental data and announcements on which a series of news reports were based. Next, it drew a compare and contrast between the news on selective mainstream media and the original information. The reported drop in the number of doctoral students could be a ‘false alarm’ for prospective target audiences, implying that digital news portals may disseminate misinformation. The analysis also revealed that the current structure of digital news making, segmented into multiformat comprising headlines, texts, videos, captions, and others, may make the information verification process more complex and obstructed for individuals. The study also points at the risk of spread of misinformation and of memory retention, amplified by the use of culture-specific symbolic numbers, which weakens our reasoning ability. The findings emphasise the importance of updating our digital media literacy and making collaborative efforts to make local research internationally sharable to advance the understanding of ‘fake news’ research in this multimedia era.

## Introduction

This study examines the authenticity of digital news coverage in the mainstream media in Japan. It highlights the mechanisms through which misinformation arises and spreads by examining a concrete case, namely ‘Doctoral Students Have Decreased by Half’.

The current case has been situated in the years 2019–2020 in Japan. Various media had reported a significant decrease in the number of doctoral students and an unpromising future for science research, followed by a new Ministry of Education measure in 2020 regarding financial support for students in Japan. The public perceived the parliament’s budget decision as a quick action taken by the government to address the situation. Meanwhile, the issue of decreasing doctoral enrollees has been an agenda item of university faculty meetings in Japan for a while. This study was therefore motivated to examine the perceived ‘gap’ that might be existent in the information between the provided and the actual state.

“Literature Review” overviews the research on ‘fake news’ in Japan and worldwide. “Methods” explains the gap analysis method used in this study. “Results and Findings” provides the findings: the first two sections, “Contextualisation of Current Graduate Programmes in Japan and Worldwide” and “Verification of the News of ‘the Decrease by Half of Doctoral Students’”, examine the original statistical information, whereas “Examination of ‘the Decrease by Half of Doctoral Students’ Breaking News” and “News Coverage Regarding Government Financial Support Measurements for Doctoral Students” examine the corresponding news coverage. The differences between the first two and the last two sections reveal the gap between the factual and the real. The phrase ‘fake news’ is used in quotes in this paper because, as will be discussed in the next section, the focus of the study is to reveal a more nuanced socio-cultural mechanism of digital media coverage, possibly a sharable worldwide phenomenon, and not to condemn specific media for their misconduct. As the research on mainstream media in Japan is scarce, the study’s findings fill a research gap, which could have a large-scale impact on various aspects of media customs and trends worldwide.

## Literature Review

This literature review is based on Scopus, Web of Science (hereafter, WoS), and Google Scholar databases. The search was conducted during December 2021 to ensure that the findings are reasonably consistent.

### General Trends of ‘Fake News’ Research

Research on ‘fake news’ has increased significantly in recent years. Article search using the keywords ‘fake news’ on Scopus and WoS produced 2295 (first article appeared in 1998 with one entry) and 2198 entries (first article appeared in 1997 with one entry), respectively. The number of entries could fluctuate depending on the search timing; however, we grasped a grand trend showing that the interest has risen sharply in the last few years. Approximately 64–65% of the entries were from the years 2020 and 2021—1476 out of 2295 entries on Scopus and 1424 out of 2198 on WoS. However, this result is limited by the fact that these databases collect information primarily in English.

The ‘fake news’ research appeals to interdisciplinary scholarship. Both Scopus and WoS provide a sophisticated analysis on the entries—the meta-analysis of the entries by category settings, such as publication periods (e.g. 2020 onwards), authors, publication types (e.g. articles, conference papers, etc.), and fields of study, which makes the laborious literature review process succinct and comprehensive. The analysis results of the field of study category showed Social Sciences, Computer Science, and Arts and Humanities on Scopus, and Communication, Computer and Information Sciences, and Social Sciences on WoS—more articles on each database in this order. The interdisciplinarity is understandable, given the nature of ‘fake news’—a kind of information flow that affects various aspects of human society. Therefore, a synthesis of different fields of study is required; the current study attempts to address this necessity.

### Definitions of ‘Fake News’

The following dictionary definition of ‘fake news’ illustrates the general public’s understanding of the term: ‘originally US news that conveys or incorporates false, fabricated, or deliberately misleading information, or that is characterised as or accused of doing so’ [1]. Several studies specifically focus on the definition of ‘fake news’. Tandoc et al. [2] executed a comprehensive review of 34 research articles on ‘fake news’ published during 2003–2017 to propose a typology of ‘fake news’ definitions with two dimensions, facticity and intention of deception, categorising them into six groups—satire, parody, fabrication, manipulation, propaganda, and advertising. Wardle and Derakhshan [3, p.5] avoid using the term ‘fake news’ owing to the complexity of the phenomenon, further categorising it into three types with two dimensions of harm and falseness:

- Mis-information is when false information is shared, but no harm is meant.
- Dis-information is when false information is knowingly shared to cause harm.
- Mal-information is when genuine information is shared to cause harm, often by moving information designed to stay private into the public sphere.

Tandoc et al. [2] further categorise ‘fake news’ based on difference in levels (higher and lower) of facticity and intention, whereas Wardle and Derakhshan [3] consider the three types based on harm and falseness as partly overlapping and depict them using a Venn diagram. The two categorisations use different terminology but similar concepts of two-dimensionality—facticity and falseness or intentionality and harmfulness. It should be noted that the focus of the current study is on ‘misinformation’—accidentally spreading false information with a lower level of harm to the audience—in the mainstream digital news media in Japan.

### Research on ‘Fake News’ in the Japanese Mainstream Media

Research focussing on ‘fake news’ and Japanese media is scarce. The number of articles found with the key concepts ‘fake news’, mainstream media, and Japan was one [4] on Scopus and two [4, 5] on WoS (including the one on Scopus), which supports the rarity of the current case study. In comparison, the number of studies on Google Scholar was 2810, but this number includes numerous types of information. To identify relevant prior studies, the search was broadened to two key concepts, ‘fake news’ and Japan; this search yielded four articles on Scopus and 20 on WoS—all articles detected by Scopus were included among those detected on WoS. Naturally, the two articles detected earlier were included in these 20 articles. The titles and abstracts of all 20 articles were examined. Additionally, full-text reading was carried out when appropriate and necessary. The examination of titles and abstracts revealed that only five articles out of 20 focus on media in Japan; the remaining 15 articles are relevant to other countries (including China, Korea, and Indonesia), with several articles focussed on the social media in these countries. The highlights of the five articles regarding Japan are summarised below.

Brown’s study [4], which is rare in that it focuses on Japan and is also the only result obtained by entering the keywords ‘fake news’, mainstream media, and Japan, analysed Russian political strategies towards Japanese media, including the mainstream having ‘a strong anti-Russian bias’ (pp.565–9). Cheng et al. [6] compared counter-balance verification mechanisms against fake news of three Asian countries, Japan, South Korea, and Thailand, using the survey method (*n* = 5218), developed arguments concerning each political system, and concluded that Japan has an anti-fake news ability fostered by democracy to respect freedom of speech. Zhao et al. [7] compared social media between Weibo in China and Twitter in Japan to report on the speed and spread of fake news by re-postings from the original (real) news. Prichard and Rucynski [8] analysed satirical news from the perspective of English as a second language, including the website Rising Wasabi (https://therisingwasabi.com/) for English-speaking residents in Japan, and reported that the estimation of ‘fake news’ as satire appeals to audiences’ critical thinking and intelligence. Furthermore, Asatani et al. [9] conducted a scientific analysis on 42 million Tweets by Japanese users and demonstrated the mechanism of information spread and imposition from a political polarisation (right and left) perspective.

The same search was also conducted on Google Scholar in Japanese using the key concepts ‘fake news’, mainstream media, and Japan. This search yielded 230 entries, which were rapidly scanned using titles and abstracts and closer reading was carried out when appropriate. Similar to the Scopus and WoS entries, among the 230 entries found on Google Scholar in Japanese, 206 were published since 2017 (88 out of 230 were published during 2020 and 2021), indicating a sudden increase in research interest in ‘fake news’ in Japan. A summary of five recently published articles that are relevant or provide some unique perspective regarding the issue is given below to present a research trend in ‘fake news’ in Japan. All articles are available via open access from their university or institutional repositories; three have English abstracts, allowing easy access to the original research.

Nishimura [10] executed survey research on 40 university students to conclude that primary news sources have been replaced by digital news sources (LINE NEWS—an application platform offered by LINE, a correspondence of WhatsApp in Japan that aggregates and distributes various digital news—and SNS) with the consequence of their failing to receive important news of higher value that more reliable print newspapers had traditionally disseminated. Namatame and Harukawa [11] has reported the results of a comparative analysis between news coverage by the two most prominent Japanese mainstream media, Yomiuri (conservative/right) and Asahi (liberal/left), in the period 2011–2019 regarding the Fukushima nuclear accident to study their opposing positions. Yonaha [12] has elucidated the spread and fixation of the belief that young people in Okinawa tend to believe fake news through an examination of 48 news media, including the 5 mainstream media (explained in the next section). Fukunaga [13], a former NHK (Nippon Hoso Kyokai or Japan Broadcasting Corporation) reporter (explained in the next section), has examined the role of mass media in rectifying and annihilating the spread of wrong information on social media, using specific examples. Furthermore, Sakamoto [14] has reported his teaching practice in media and information literacy classes, arguing the necessity of a habit of fact checking against fake news and disinformation to maintain a democratic society.

Research by Namatame and Harukawa [11] and Fukunaga [13] can be said to share some aspects with the current case study in that each focuses on misinformation by the mainstream media on a specific news topic. However, the current case study covers recent and novel aspects of news coverage. The previous two studies primarily analysed print newspapers, whereas the current study focuses strictly on digital media.

### Unique Features of Japanese Mainstream News Media

The unique status of the mainstream media in Japan—major (large circulation), main (high authority), and mass (large population)—is notable. Considering this fact is important because the following case study takes examples from digital news portals of Asahi, Yomiuri, Nikkei, Yahoo!, and NHK, all of which are mainstream in Japan. Additionally, the database search on Scopus and WoS reveals that each article may use the term ‘mainstream media’ in different ways that is we may analyse different ideas under the same category to deduce a conclusion.

Traditionally, four major daily newspapers—Asahi, Mainichi, Nikkei, and Yomiuri, presented in alphabetical order—have been considered ‘mainstream’ press due to their large-scale print and paid circulation (sometimes including Sankei, making them the most significant five) [15, 16]. Notably, the volume of circulation of these domestic ‘mainstream’ newspapers has been unexpectedly large, with Yomiuri occupying the first place internationally and Asahi the second, followed by USA Today [17, p.58, 18]. However, the industry has been rapidly shifting from print-based to multimedia-based. It is notable that all four big mainstream newspapers also provide English versions of the news portals for topics with international appeal. Therefore, with the relatively large population of the country, the social impact of local Japanese mainstream news media on people’s notions and behaviours could be huge, potentially sharable, and influential worldwide.

The status of Yahoo! News and Google News in this territory should also be mentioned, though both are outside our traditional notions of mainstream news media. The Japanese versions of Yahoo! News (https://news.yahoo.co.jp/) and Google News (https://news.google.com/), which are also accessible as smartphone applications, have rapidly joined the major and popular digital news portals for information acquisition and circulation. These vary from the previously mentioned ‘mainstream’ press in that they are primarily aggregators of news from other news portals. A Japanese version of Yahoo! News has started to post original news, which indicates that there should be locally specific features among different versions of Yahoo! News in other countries. In addition, the current case study includes examples from NHK (https://www.nhk.or.jp/), Japan’s single public broadcaster since March 1925, being a radio broadcasting station [19] roughly corresponding in status and role to the BBC in the UK and CNN in the US. NHK has now become a multimedia news portal, with an application accessible on smartphones that is becoming a popular information source in Japan, similar to Yahoo! and Google News in many other countries worldwide.

## Methods

To analyse the process of the occurrence of misinformation, this case study takes the following four steps: (1) contextualisation of graduate programmes in Japan compared with the state of the world, (2) verification of doctoral enrolment using ministerial data, (3) chronological examination of the news coverage of the decrease in the number of graduate students, and (4) examination of the ministerial support measures and news coverage of the issue regarding stakeholders.

The analysis method applied in this case study follows a ‘reversed gap analysis’—a methodology to examine the actual state and the desired/expected state for improvements [20, 21] but in a reversed way—to elucidate the possible gap between the factual data and the actual news. Steps (1) and (2) are aimed at obtaining the factual data, whereas steps (3) and (4) assess how the relevant news was reported and developed. For steps (1) and (2), relevant original ministerial/governmental reports were detected and closely examined, with the reference information captioned/footnoted in the news; for steps (3) and (4), relevant publicly accessible online news with particular focus on mainstream media were identified via a keywords search (‘doctoral students’ and ‘50% decrease’) and examined.

The focus on the Internet as an information source in this study was based on the trends of media use for the relevant audience. The annual Survey Report on Usage Time of Information and Communications Media and Information Behaviour [22] states that Internet portal news sites have become the primary news sources followed by television, paper-based news, and radio for individuals in their 20 s and 30 s, the primary target audience for this case study in Japan (see “Contextualisation of Current Graduate Programmes in Japan and Worldwide”).

## Results and Findings

### Contextualisation of Current Graduate Programmes in Japan and Worldwide

This section provides the factual and statistical background information on graduate education in Japan, on which the subsequent analyses were developed. Table 1 outlines the number of graduate students as of May 2020, before the effects of COVID-19, enrolled in a master’s or doctoral programme in an academic year (AY) [23]. ‘Master’s’ and ‘doctor’s’ include different fields of study. In Japan, research degrees and practical degrees are distinguished; that is within a particular discipline, such as medicine and law, both research degrees (PhD) and so-called ‘professional degrees’ could exist. In Table 1, the numbers in ‘master’s’ and ‘doctor’s’ only include research degrees. An independent ‘professional master’s’ category for professional fields, such as law, business, and teacher training, has emerged in Japan since 2003 to meet new global demands [24]. The numbers in Table 1 include international students as of AY 2020 (total, 10,471; males, 4920; females, 5551) [23].

**Table 1:** Numbers of graduate students in Japan, AY 2020

	Master’s			Doctor’s			Professional Master’s		
	Total	Males	Females	Total	Males	Females	Total	Males	Females
Entry	71,954	49,861	22,093	14,659	9,984	4,675	8,103	5,337	2,766
Total	160,297	109,364	50,933	75,345	49,757	25,588	18,887	12,426	6,461

According to the most recent Organisation for Economic Co-operation and Development (OECD) data of the rate (%) of entry into graduate programmes (Level 7 or master’s equivalent), Japan ranks 29th (males 23rd, females 33rd) amongst 37 countries as of 2018 [25], followed by the US (ranked 30th) and South Korea (ranked 31st). OECD data vary in the treatment of local and international students’ enrolment, and in the case of Japan, it includes international students. Therefore, the rate of entry into graduate programmes is not necessarily high amongst OECD countries, similar to that of two countries with which Japan has the highest rate of trade (alongside China) [26].

**Fig. 1:**
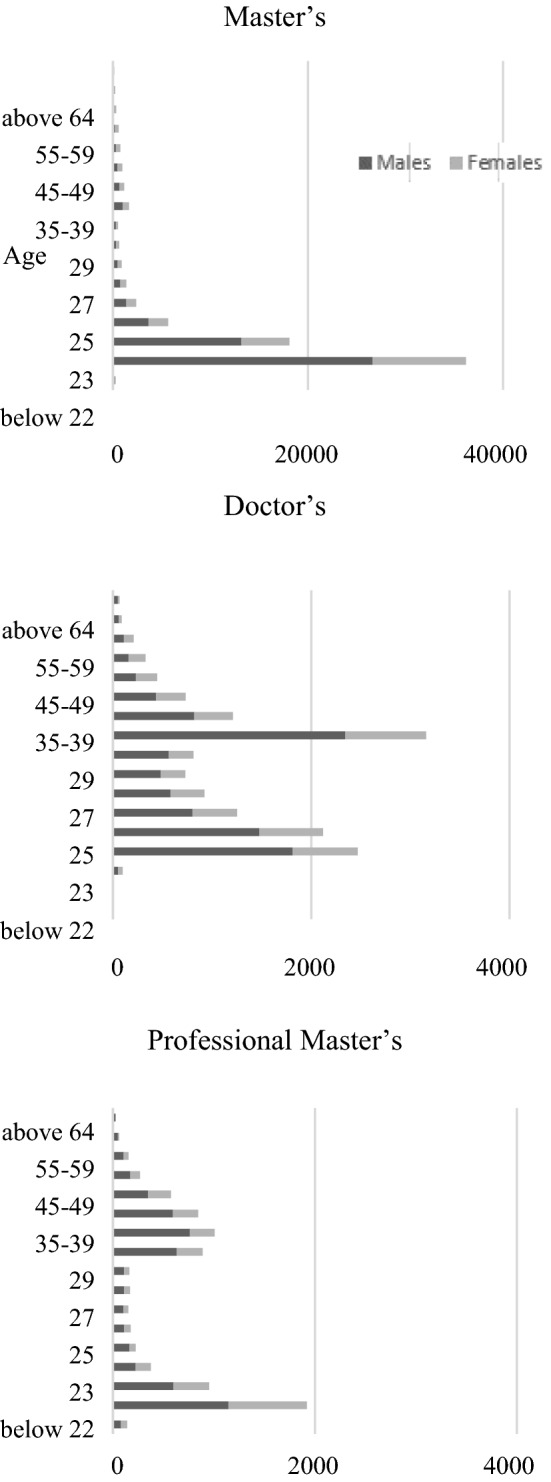
Numbers of enrolment for graduate programs by category, age, and gender in 2020

**Fig. 2:**
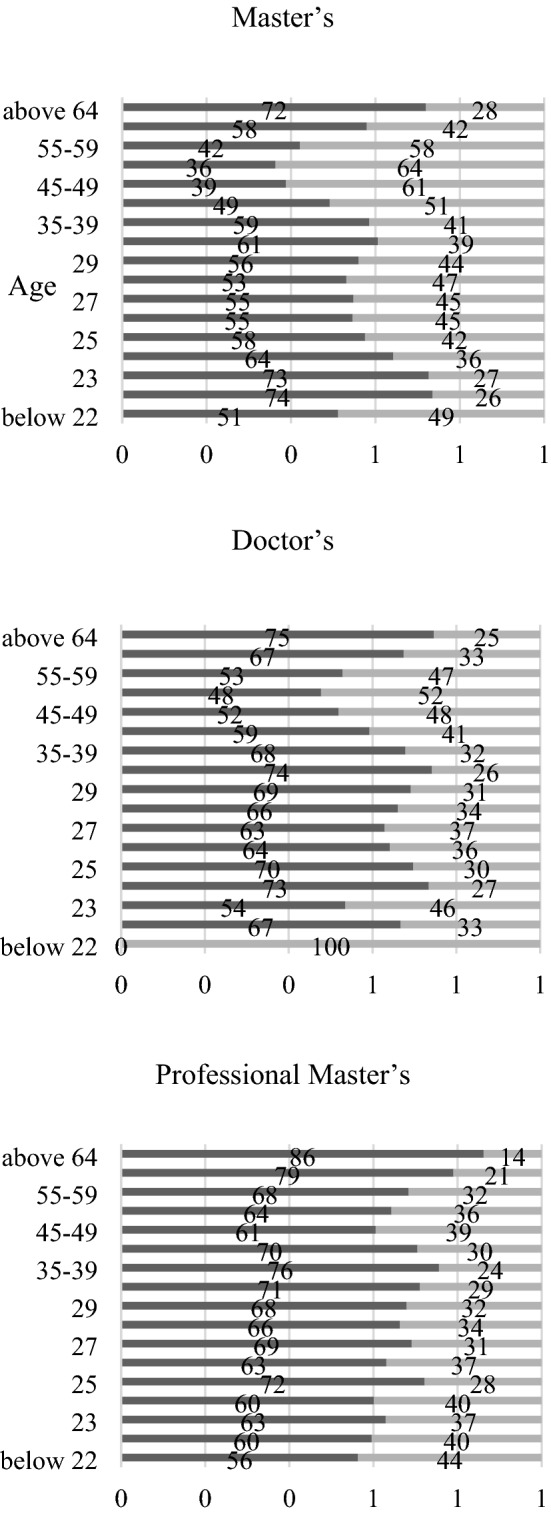
Gender ratios of graduate student enrolments by age in 2020 (dark grey: males, light grey: females)

For the analyses in “Literature Review” and “Methods”, Figs. 1 and 2 depict the breakdown of students by age and gender from the School Basic Survey 2020 data [23]. Figure 1 presents the enrolments for master’s and doctor’s degrees by gender and age group at five-year intervals; Fig. 2 presents the gender ratios by age group for Fig. 1. Given that the number of doctorates is much smaller, the unit for master’s takes the maximum value of 40,000, whereas that for doctoral and professional master’s takes the maximum value of 4000—one-tenth—in Fig. 1 to improve the visual compatibility.

The data indicate a stunning trend of graduate programmes in Japan that may not have been publicly recognised. First, as shown on the left, students enrolled in master’s programmes reach a peak at approximately 22–23 years of age. In contrast, for doctoral programmes, two peaks are observed, first at 24, presumably right after the master's programme, and the second at 30–34, presumably after certain life experiences, including work. This observation is tentatively called an ‘M-shaped’ phenomenon. Moreover, as mentioned in the “Methods” section above, the analysis of digital news portals has higher validity as they are the most preferred media for the 20s and 30s. Second, the figures in the right panel present the gender distribution by age for each level of programme; both master’s and doctoral programmes follow a similar pattern, that is they have more male students for the younger, middle-age, and senior age groups. This observation is tentatively named the ‘W-shaped’ phenomenon from the male enrolees’ perspective, which simultaneously represents the ‘M-shaped’ phenomenon from the perspective of female enrolees. Third, for professional master’s, the enrolment is more visible for male students and their numbers increase as they become older.

### Verification of the News of ‘the Decrease by Half of Doctoral Students’

In the news analysis in “Examination of ‘the Decrease by Half of Doctoral Students’ Breaking News” and “News Coverage Regarding Government Financial Support Measurements for Doctoral Students”, two reports, one from the Ministry of Education and another from a research team at the University of Tokyo, have frequently been cited as the information sources for media coverage. A closer examination reveals that both could have come from different chapters/years of the same Japan Science and Technology Indicators by the National Institute of Science and Technology Policy (NISTEP) reports [27].

**Fig. 3:**
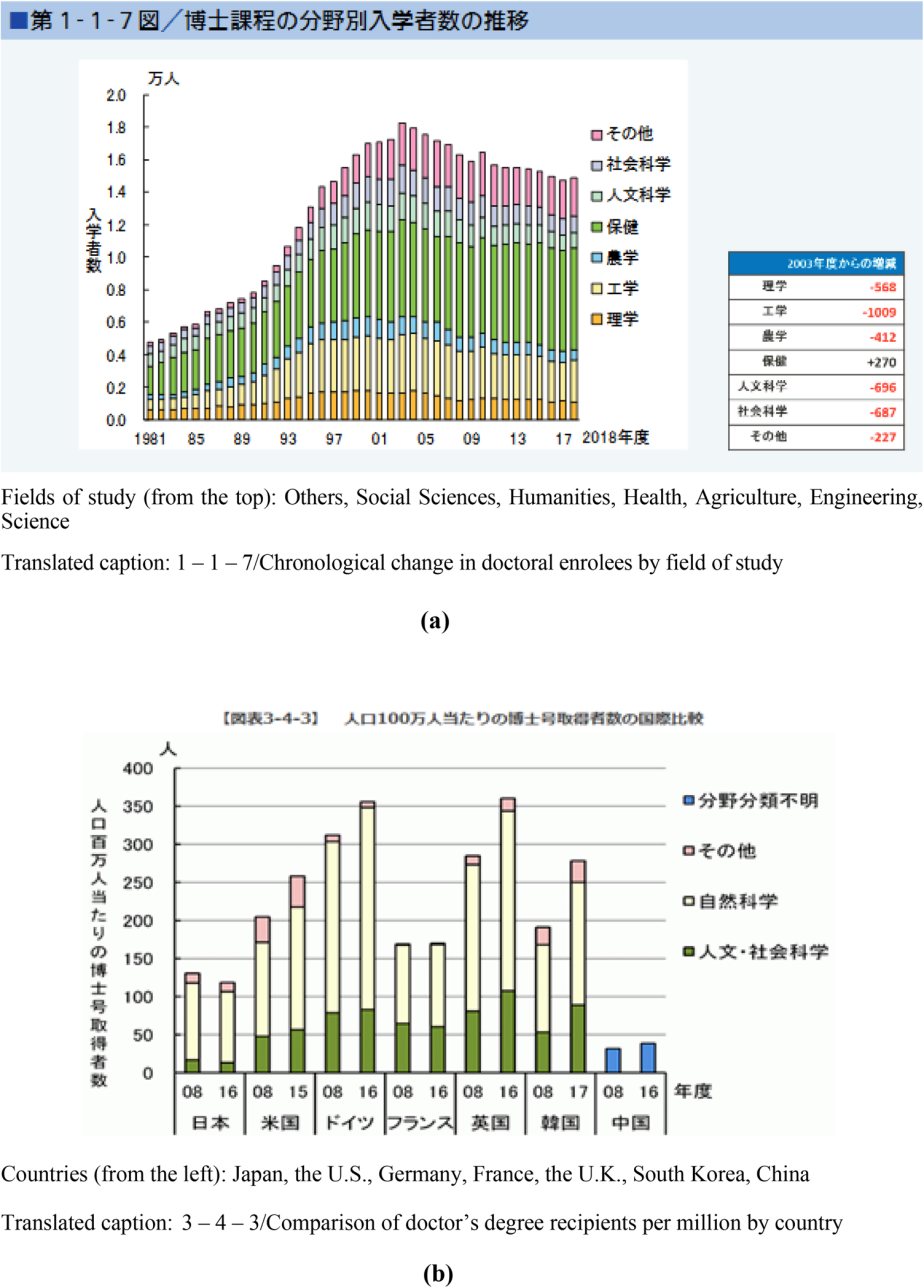
**a** State of doctoral students in Japan, 2019 (NISTEP, annual). **b** State of doctoral students in Japan, 2019 (NISTEP, annual)

Figure 3a is the reference for the reports of the doctoral ‘decrease by half’ news; the data come from further back in the Ministry of Education, Culture, Sports, Science, and Technology (MEXT) School Basic Survey 2003–2018. The graph is based on the peak of enrolees in AY 2003 (18,232 enrolees), which dropped to 14,903 in AY 2018, an 18.3% decrease. Limiting the numbers to the combined science and technology majors—the number in AY 2003 being 5221 (1650 + 3571 enrolees) and that in AY 2018 being 3644 (1082 + 2562)—shows a 30.2% decrease. Meanwhile, the number of adult and international students out of the total enrolees decreased from 11,637 (18,232–3952–2643) in AY 2003 to 4526 (10,168–4274–1368) in AY 2018, showing a 61.1% decrease. This trend seems to have continued in AY 2019, at 5963 (14,976–6349–2664), which is a 48.3% decrease, and in AY 2020, at 5,703 (14,659–6335–2621), a 51.0% decrease since the peak year 2003. Ultimately, the reports of a ‘decrease by half of doctoral students’ are likely to refer to the statistics *excluding* adult and international students.

Figure 3b indicates the number of PhD recipients in Japan compared with some other countries. For the consistency of the arguments/analyses, the same year’s data (2019 indicator) are presented. The raw figures are available from the NISTEP website. In Japan, the number of PhD recipients decreased from 131 in 2008 to 118 in 2016 per million people. NISTEP’s addendum notes that the data come from the 2016 MEXT report, made public in 2019 [28]. Therefore, both figures are ultimately from the same MEXT School Survey data. The reason for the three-year delay in publication is unknown.

### Examination of ‘the Decrease by Half of Doctoral Students’ Breaking News

Based on the review in “Contextualisation of Current Graduate Programmes in Japan and Worldwide” and “Verification of the News of ‘the Decrease by Half of Doctoral Students’”, three breaking news stories from major news portals are chronologically listed to be examined for information accuracy. Since the original news contents were in Japanese, an English translation of the main points of the coverage in focus is presented below.

#### Headline Gist: ‘Doctoral Candidates Reduced by Half, Zero Nobel Prize Winner Era Approaching’ by Toyokeizai.net (2019/09/30) [29]

This article highlights graduates’ need for social and financial support. Although the headline gives a vague impression of ‘half decrease’, the article develops its examination to accurately detail the half reduction referring to the number of adult students with the reasoning that ‘adult students are less likely to become researchers’. The article mentions ‘the real number of doctoral students in science fields decreased to half’ compared with 15 years ago; however, as of 2019 (referring to AY 2018 data), ‘the number in science and engineering fields’ experienced an approximated 30% decrease, not 50%, as examined above.

#### Headline Gist: ‘Japanese Companies Stultify Doctors, PhD Recipients Decreased by 16% over 10 Years’ by Nikkei.com (2019/12/8) [30]

This article highlights Japanese companies’ call for more effective use of PhD holders’ specialisations. The news sources are cited as NISTEP and MEXT. The description of Japan’s annual PhD ‘recipients’ having decreased by ‘16%’ from 2006 to 2016, in contrast to the numbers in ‘European and American countries’ having a ‘two-digit’ increase, could be misleading.

**Fig. 4:**
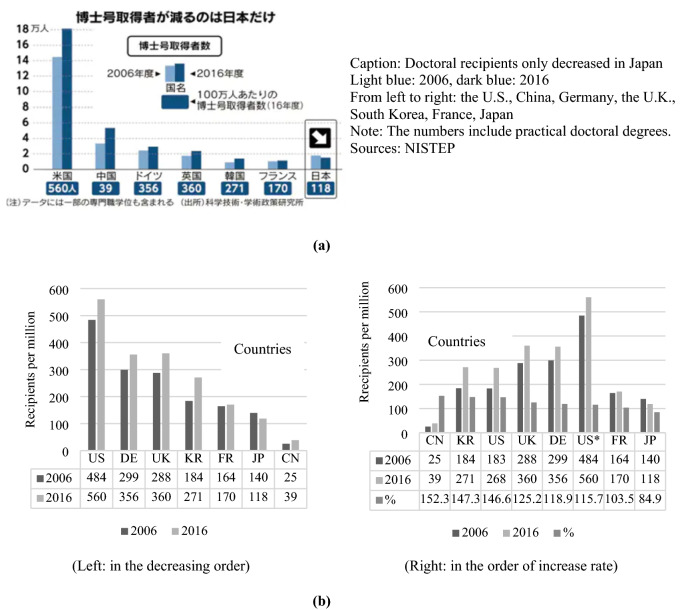
**a** Doctorate and doctoral recipients per million by country in 2006 and 2016 (NISTEP from the Nikkei news portal). **b** Doctorate and doctoral recipients per million by country in 2006 and 2016 (from the original MEXT data)

Figure 4a and b are juxtaposed to make the ambiguity more visible. Figure 4a has been taken from the news, and Fig. 4b is created from the original NISTEP data. Two bars represent the US in NISTEP 2019 in the figure on the right because the US publicises research PhDs and other types of doctors, including ‘first-professional degrees’, such as for medical and law professionals [31]. From 2006 to 2016, the change rate is also calculated for this analysis as a percentage indicated by the right-most bars in Fig. 4b. These confirm the news information that only Japan experienced a decrease in the number of doctorate recipients; however, if the main point of the data analysis concerns the increase in PhD recipients, the increase seems to be the highest in China and South Korea, followed by the US, the UK, Germany, France, and Japan, in this order. Furthermore, the increased percentage of 103.5 in France does not fit the description of a ‘two-digit’ increase.

In addition, the news is presented in several formats, including webpages, Instagram, or other social media posts, and television broadcastings of 10-min format. Although the main point of the decrease in the number of doctorate recipients is retained in all news formats, the audience could miss critical details with obscure source information due to the format, potentially ending up altering their perception with quasi-false information.

#### Headline Gist: ‘The Number of Students in the Doctoral Programmes in Science Decreased to Half, Compared with its Peak’ by NHK (2020/10/4, 19:56) [32]

The main point of this 4-minute web news is the necessity of financial and employment support for doctoral students endorsed by the 2019 Nobel laureate Dr Yoshino. The news link was found to be inaccessible when writing this paper, which is an excellent example of the fragility of video-based online news. Fortunately, the data have been conserved and was available for analysis. The run time underneath the figures provides specific information about the length of the news segment and the time when these figures appear.

**Fig. 5:**
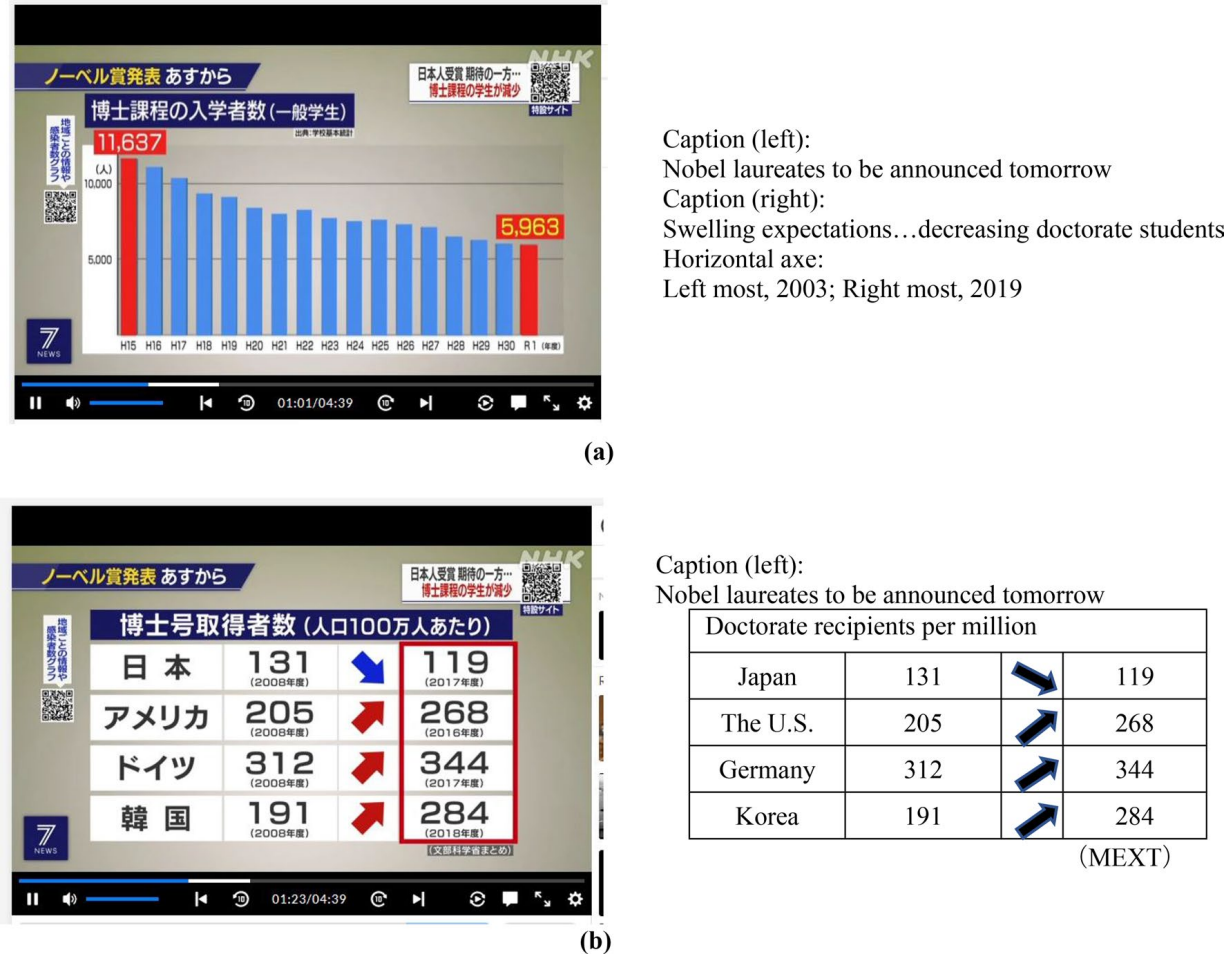
**a** Chronological change in the numbers of doctoral enrolees, 2003–2019. **b** Chronological change in the numbers of doctorate recipients, 2008 and 2017/2018

Figure 5a and b is screenshots from the news to demonstrate the content. Both data sets have been taken from MEXT in the news. The specific figures in the data assume that they are based on MEXT AY 2019, excluding adults and international students, with a reference to ‘general students’ in the parentheses, which is a similar estimation as that presented in “Methods”. However, non-expert audiences may not identify the sources. As shown in Fig. 5b, the news says that “compared with the US, Germany, and South Korea, ‘the level of Japan’ has dropped by ‘less than half’’’. It is not clear from the narration who decides the level, but this could be inferred as 119 × 2 = 238 per million people in Japan, which is ‘less than half’ of the numbers in other countries per million represented in the figure—268 in the US, 344 in Germany, and 284 in Korea—as an interpretation. Interestingly, they seem to follow a similar estimation as discussed previously; the 50% decrease refers to ‘general students’, excluding students of specific categories.

With regard to ‘general students’ and the pure PhD data, this broadcast could be considered more factual with a higher level of verification of the original data sources. Nearly one year has passed since the initial news was disseminated by the Toyo Keizai news portal in September 2019, as examined in “Headline Gist: ‘Doctoral Candidates Reduced by Half, Zero Nobel Prize Winner Era Approaching’ by Toyokeizai.net (2019/09/30) [29]”. Paradoxically, the news presented in “Headline Gist: ‘The Number of Students in the Doctoral Programmes in Science Decreased to Half, Compared with its Peak’ by NHK (2020/10/4, 19:56) [32]” leads us to conclude that the previous news regarding this issue was not published before it had been sufficiently verified factually.

### News Coverage Regarding Government Financial Support Measurements for Doctoral Students

This step verifies the news regarding the governmental aid plan to cope with the doctoral students’ unprivileged situation, which was announced nearly two years after the initial digital news by Toyo Keizai in September 2019. Specifically, on 15 December 2020, a financial support measurement for doctoral students was announced through a letter by the MEXT [33]. The letter announced support for 15,000 ‘persons’ (not specified) to accomplish the target of ‘research fortification and young researcher support total package’. This section examines a series of four digital news with different interpretations; all news items were published within five days of this announcement.

#### Headline Gist: ‘The Government Provides Financial Support of 2,300,000 Yen in Annual Aid to 1,000 Doctoral Students in Advanced Fields’ by Yomiuri.co.jp (2020/12/15) [34]

It is not clear why this news specifies the relevant party to be 1,000 doctoral students. The news also details that ‘the students who advanced to doctoral programmes are about 6,000 in 2018 or the half over 15 years’ without a clear source information, which makes verification more difficult. Nevertheless, the estimation of ‘half’ refers to the change in 15 years from 2003 to 2018; this estimation may have been similar to that of NHK news as discussed in “Headline Gist: ‘The Number of Students in the Doctoral Programmes in Science Decreased to Half, Compared with its Peak’ by NHK (2020/10/4, 19:56) [32]”, which was broadcast on 4 October 2020.

#### Headline Gist: ‘MEXT Supports Half of the Students Advancing to the Doctor Programmes’ by Yahoo! News Japan (2020/12/16) [35]

The news states in the main text that ‘the support is aimed at 15,000 students covering half of the students who advance from master’s to doctor’s programmes...the students in the doctor’s programmes represent a total of 75,000’. This news does not cite any specific source of information, which makes verification extremely difficult. The reference of 15,000 doctoral students having advanced from mater’s programmes is not verifiable [36]. The MEXT data as of 2020 gives the total number of master’s degree recipients as 73,169, among whom those who advanced to doctoral programmes are 6,714. The reported total number of students in doctoral programmes as 75,000 is assumed to be a rounded estimation of the MEXT data of the same year, that is 74,711 [37].

#### Headline Gist: ‘2,500,000 Yen Annual Aid to 1,000 Doctoral Students in Advanced Fields’ by Asahi.com (2020/12/20) [38]

This news headline is the closest to that examined in “Headline Gist: ‘The Government Provides Financial Support of 2,300,000 Yen in Annual Aid to 1,000 Doctoral Students in Advanced Fields’ by Yomiuri.co.jp (2020/12/15) [34]”, except that the amount of financial aid slightly differs (2,300,000 yen and 2,500,000 yen). The news article cites ‘MEXT representative(s)’ as its source and reports on the decrease in the number of master’s students directly advancing to doctoral programmes (‘9.3% in 2018’, which is factual), the difficulty faced by ‘PhD recipients’ in finding jobs with ‘only 70%’ being hired or retained for research, and the decrease in the number of academic paper publications due to ‘the decrease in the number of young researchers’. However, the number of doctoral programme graduates regardless of completing the degree is 69.8%, as per the data by MEXT in 2020; but when the number of all-but-dissertation graduates is excluded, the percentage could currently be as high as 92%. In addition, the MEXT representative(s)’ reference to the decrease in the number of academic outputs ‘linked to the decrease in the number of young researchers’ likely refers to the MEXT 2018 report [39]; however, the causal link between the decrease in the number of academic papers and the decrease in the number of young researchers cannot be established from the MEXT report itself.

#### Headline Gist: ‘2,900,000 Yen Annual Aid to 7,000 Doctoral Students’ by NHK (2020/12/20) [40]

This article refers to the decrease in the number of master’s students directly advancing to doctoral programmes and the decrease in the number of research article publications from Japan in the natural science fields, mentioning that doctoral students write 20% of the papers. The information was vaguely credited to MEXT. NHK news may be more factual in its efforts to trace back the MEXT summative information to the original data compared to the article examined in “Headline Gist: ‘2,900,000 Yen Annual Aid to 7,000 Doctoral Students’ by NHK (2020/12/20) [40]”. However, if the statement about 20% of the papers comes from another report by NISTEP [41], initially published in 2010 (approximately 10 years ago), the news is referring possibly to the situation a few years before 2010, given the time required for data collection and the formal publication process.

## Implications

This study investigates the prevailing reports of a ‘decrease by half of doctoral students’ in Japan as a case for examining the authenticity of digital news in mainstream media by comparing the original information and the news coverage. The process revealed that the media coverage could convey its emphasis, which could sometimes slightly differ from the sources and develop independently. The process also identifies the traces of efforts made by the media to make the news factual; usual readers and users of digital media cannot easily verify the truthfulness of such news.

This study is novel in the following ways: (1) the provision of research review, including English and Japanese media, to clarify the location of the current study, (2) the elucidation of the possible spread of misinformation in the mainstream media in its findings, and (3) its particular focus on the trends of the doctoral student population in Japan as a target case study. This study also attempts at a wider scholastic sharing of a local social phenomenon, which, although studied in the non-English context of Japan, potentially has a more comprehensive application and relevance to international social concerns.

A review of prior research and the findings of the current study also point at directions to be explored in future research. The first point is related to the current multiformat news delivery structure. Due to digitalisation, the media has gained the capacity to disseminate the same news content in various formats, not only their leading news portal websites and prints but also segmented into numerous platforms and applications, such as Instagram, LINE, and Twitter. These present a distinct method of presenting news wherein the headlines convey the main points with supporting graphics, only displaying brief, text-based information. The current study confirms that the actual news content could be understated in this process, and the source information could be lost. As exemplified by the current study, fact-checking [14] could be a laborious task demanding particular skills and knowledge, which, even if acquired by the lay audience at some point in their lives, may not be adequately applied in this situation. Moreover, the fragmentation of news content into various media formats is likely to contribute to making their appearance on the small smartphone screens and ‘mainstream media’ news indistinguishable.

The second point concerns the distinction between misinformation and disinformation. As mentioned in the “Literature Review” section, though both include some degree of false information, the predominant difference lies in the intention of causing harm to the information receivers. If the series of news outputs are linked to the governmental decision on financial aid, it could fall into the category of ‘propaganda’ [2, p.10] as a function to support the policy. However, the phenomenon analysed in this study belongs to a different category because of its presumed unintentionality and severe attitude to trace; moreover, the news contents in this study are consistent with the original governmental announcement. However, a further trace after completion of the current analysis confirmed that in reality, the financial aid is likely to be for the 24–25 students currently in a master’s programme in specific limited fields of information, artificial intelligence, quantum technology, and materials limited to 55 specific universities [38]. This line of thought raises another issue: some readers who may have made a quick decision to enrol in doctoral programmes based solely on this piece of news may have committed a severe miscalculation. In this sense, if, as concluded, ‘no empirical estimates of the exposure to fake news stories through mainstream news media exist’ [42, p.168], this case study might have provided some, as many of the examined news stories were taken from the mainstream news media in Japan.

The third point addresses the relative scarcity of ‘fake news’ research in the mainstream media. As described earlier, the Japanese mainstream media enjoy special status and function because of their threefold characteristics of being major, main, and mass. Unlike research in social media, where vast data could be retrieved for anonymous analysis [12], the influence and power of mainstream media in Japan, with its political positions, is far more significant than those in other countries in the Japanese territory. People may tend to avoid touching this topic for any possible adverse effects on themselves. This point demands a much more refined analysis, but the Japan-based article search on Google Scholar gave the impression that more female scholars might be active with this specific research topic than their male counterparts. This tendency may be related to female scholars’ relative lower commitment to the establishment and authority. Whether this is the case and whether this tendency is somewhat internationally sharable is also a possible avenue for future research. A more practical reason could also be the difficulty of transferring the information first appearing in the Japanese language into some other language that is internationally sharable, including English. This study was possible because the core information used here is based on figures that are easily transferable to universal standards.

The fourth point concerns unexpected findings regarding the recent trends of graduate-level enrolments in higher education in Japan (Sect. “Contextualisation of Current Graduate Programmes in Japan and Worldwide”). The factual information was also verified as part of the findings, tentatively named ‘M-shaped’ and ‘W-shaped’ phenomenon. ‘M-shaped’ or the existence of the two waves of 22–23 and 30–34 age groups can be most relevant to the target audience for the news information regarding graduate-level education in Japan. These support the validity of the current study’s focus on the digital news medium and also provide directions for higher education management and policy making. It will also be more effective and efficient to divert the graduate-level curriculum into two branches of young graduates and those of slightly advanced age as the needs and demands of the latter shall be different from the former, given the greater life experiences of the latter before re-entry to higher education. For mature students, minimise the duplication of their already acquired knowledge and skills and maximise the activation of their life knowledge and skills. Moreover, further investigation into the reasons behind the ‘W-shaped’ phenomenon or the reversed relation between the male and female students at different life points in terms of graduate-level entry—puzzlingly, in the governmental statistics, only the numbers of ‘male’ students and the total are available so we had to extract the number of males from the total to gain the number of females for all the datasets—may help unveil the socio-cultural aspects of our society.

The last point is relevant to accepting new information and memory retention, which could be relevant to diffusion of propaganda. In the current case study, the figure of 50% has been repeated to the extent of it becoming a symbol of the main idea of the news content, namely doctoral students are 50% less, therefore, support 50% of them. In Japan and possibly some neighbouring countries, numbers divisible by 5, such as 5, 10, 25, 50, and 100, are considered excellent numbers as they are easy to recognise, accept, and memorise. The retention of wrong information can become easier when the information is spread by the mainstream media [42]. Incidentally, the number 666 might have caught people’s attention when referring to the number of infected populations [43–45] amid the pandemic—this symbolic number is strongly associated with the devil and the evil originating from biblical stories. The use of specific numbers over a series of news coverage could be mentally disturbing, lowering one’s mental ability to engage in everyday reasoning and information verification. With regard to this, we may bring students’ attention in digital literacy classes to these effects to enable them to avoid falling into unwanted information pitfalls.

Finally, as was partly demonstrated in the literature review using Google Scholar in Japanese (Sect. “Research on ‘Fake News’ in the Japanese Mainstream Media”), research worth international sharing but published only in a local language may abound in other local languages as well. In such case, further research on the trends of mainstream digital media in different local languages should ideally be made to advance various relevant fields of study in the future.

## Conclusion

This study highlighted a unique aspect of misinformation in Japanese news media and society, illustrated using a specific case. The probable effects could be even more severe as none of the relevant people involved in the process has any apparent malice to cause but rather a belief to support and help other people from a place of goodness. A void in media coverage research regarding Japanese mainstream media, which represents the 3Ms (major, main, and mass) at the international scale, could be a significant gap in research. The reason for social news media studies outnumbering the mainstream news media research worldwide may partly be the inaccessibility to the news coverage originating in local languages. In this case, we hope that this small case study serves as an example to illustrate that we can overcome this hurdle, demonstrating that even if the work becomes laborious it would be equally rewarding.

## References

[1] Oxford English Dictionary (OED). Fake news. https://www.oed.com/view/Entry/67776?redirectedFrom=fake+news#eid1264306660. Accessed 21 Dec 2021.

[2] Tandoc EC, Lim ZW, Ling R (2018). Defining ‘fake news’: a typology of scholarly definitions. Digit J.

[3] Wardle C, Derakhshan H. Information disorder: Toward an interdisciplinary framework for research and policy making. Council of Europe. 2017; p. 27.

[4] Brown JD (2021). Russian strategic communications toward Japan: a more benign model of influence?. Asian Perspect.

[5] Ross AS, Rivers DJ (2018). Discursive deflection: accusation of “fake news” and the spread of mis-and disinformation in the tweets of President Trump. Social Media + Society..

[6] Cheng JW, Mitomo H, Kamplean A, Seo Y (2021). Lesser evil? Public opinion on regulating fake news in Japan, South Korea, and Thailand–a three-country comparison. Telecomm Policy.

[7] Zhao Z, Zhao J, Sano Y, Levy O, Takayasu H, Takayasu M, Li D, Wu J, Havlin S (2020). Fake news propagates differently from real news even at early stages of spreading. EPJ Data Sci.

[8] Prichard C, Rucynski J (2019). Second language learners’ ability to detect satirical news and the effect of humor competency training. TESOL Journal.

[9] Asatani K, Yamano H, Sakaki T, Sakata I (2021). Dense and influential core promotion of daily viral information spread in political echo chambers. Sci Rep.

[10] Nishimura K (2020). Less informed citizens in the results of thriving online news media: survey results to confirm the logic of democracy crisis. Appl Sociol Res Rev, Kinki University.

[11] Namatame N, Harukawa M (2020). Study of eight-year media coverage regarding the accident of Fukushima Daiichi Nuclear Power Plant: on health issues of the local population. Bull Tohoku Fukushi University.

[12] Yonaha S (2020). How newspaper discourse of the association between fake news and youth spread. Socio-Inform.

[13] Fukunaga H. Research report: misinformation, disinformation, and media's counter news in the age of social media: discussing matters to be noted. The NHK Monthly Report on Broadcast Research. 2019;69(8):100–10. 10.24634/bunken.69.8_100.

[14] Sakamoto J (2018). The possibility of fact checking practice in media and information literacy education. Bull Faculty Lifelong LearnCareer Stud.

[15] The Japan Audit Bureau of Circulation: Circulation Report (First Half of 2021). The Bunka News. 2021. https://www.bunkanews.jp/article/237791/. Accessed 6 Aug 2021.

[16] Suzuki T, Kanou E, Arakawa Y. (2013, November). Comparative analyses of textual contents and styles of five major Japanese newspapers. In Proceedings of the 27th Pacific Asia Conference on Language, Information, and Computation, PACLIC 27, pp. 450–8.

[17] World Association of News Publishers. WAN-IFRA's World Press Trends 2016 report. https://wan-ifra.org/insight/world-press-trends-report-2016/. Accessed 22 Dec 2021.

[18] What is the #1 newspaper in the world? morethingsjapanese.com. 2019. https://morethingsjapanese.com/which-country-has-the-highest-newspaper-circulation-in-the-world/. Accessed 22 Dec 2021.

[19] Nippon Hoso Kyokai (NHK). NHK corporate info: History. https://www.nhk.or.jp/corporateinfo/english/history/. Accessed 20 Dec 2021.

[20] The complete guide to gap analysis. https://www.smartsheet.com/gap-analysis-method-examples. Accessed 21 Dec 2021.

[21] Van Auken S, Chrysler E, Wells LG, Simkin M (2011). Relating gap analysis results to information systems program attitudes: the identification of gap priorities and implications. J Educ Bus.

[22] Ministry of Internal Affairs and Communications (MIC). Survey report on usage time of information and communications media and information behavior 2020. https://www.soumu.go.jp/iicp/research/results/media_usage-time.html. Accessed 22 Dec 2021.

[23] Ministry of Education, Culture, Sports, Sciences and Technology, Japan (MEXT). Basic School Survey 2020. https://www.e-stat.go.jp/stat-search/files?page=1&toukei=00400001&tstat=000001011528. Accessed 22 Dec 2021.

[24] MEXT, Professional graduate schools. https://www.mext.go.jp/a_menu/koutou/senmonshoku/index.htm. Accessed 22 Dec 2021.

[25] Organisation for Economic Co-operation and Development (OECD). Advance rate to graduate schools. 2018. https://www.globalnote.jp/post-14176.html. Accessed 22 Dec 2021.

[26] Trade Statistics of Japan. Top 10 import and export countries of Japan since 1995–2019. 2019. https://www.customs.go.jp/toukei/suii/html/time_latest.htm. Accessed 22 Dec 2021.

[27] National Institute of Science and Technology Policy (NISTEP). Japanese Science and Technology Indicators. (Annual). https://www.nistep.go.jp/research/science-and-technology-indicators-and-scientometrics/indicators. Accessed 22 Dec 2021.

[28] MEXT. The numbers of degree recipients. 2019. https://www.mext.go.jp/component/a_menu/education/detail/__icsFiles/afieldfile/2019/06/17/1299723_13.pdf. Accessed 22 Dec 2021.

[29] Iwamoto N. The number of doctoral candidates reduced in half, zero Nobel Prize winner era approaching. 2019. https://toyokeizai.net/articles/-/304852. Accessed 22 Dec 2021.

[30] Kitazume T, Ogawa M, Oikawa A. Japan companies stultify doctors, PhD holders 16% decreased over 10 years. 2019. https://www.nikkei.com/article/DGXMZO53006550V01C19A2SHA000/. Accessed 22 Dec 2021.

[31] U.S. Network for Education Information (USNEI). Structure of the U.S. education system: first-professional degrees. (Last modified 22 Feb 2008). https://www2.ed.gov/about/offices/list/ous/international/usnei/us/edlite-structure-us.html. Accessed 22 Dec 2021.

[32] NHK. Doctoral students who support a science-and-technology-based country decreased half from its peak. 4 Oct 2020. https://www3.nhk.or.jp/news/html/20201004/k10012648001000.html. (Became inaccessible on 22 Dec 2021 but the same graphics are reused on 8 Dec 2020, at https://www3.nhk.or.jp/news/special/nobelprize2020/article/article_01.html). Accessed 22 Dec 2021.

[33] MEXT. To students who aim to pursue doctors. 2020. https://www.mext.go.jp/content/20201215-mxt_jyohoka01-000011639_01.pdf. Accessed 22 Dec 2021.

[34] Yomiuri.co.jp. Original coverage: The government supports 2,300,000 yen annual aid to 1,000 doctoral students in advanced fields. 2020. https://www.yomiuri.co.jp/national/20201215-OYT1T50084/. Accessed 22 Dec 2021.

[35] Murohashi Y. MEXT supports the half of students advancing to the doctor programs. 2020. https://news.yahoo.co.jp/byline/murohashiyuki/20201216-00212760/. Accessed 22 Dec 2021.

[36] MEXT. 79 Number of graduates by status of master’s course (4–1). Basic School Survey 2019. 2020. https://www.e-stat.go.jp/stat-search/files?page=1&layout=datalist&toukei=00400001&tstat=000001011528&cycle=0&tclass1=000001135783&tclass2=000001135810&tclass3=000001135818&tclass4=000001135821&tclass5val=0. Accessed 22 Dec 2021.

[37] MEXT. 5 Number of graduate students by graduate school (3–2) Basic School Survey 2019. 2020. https://www.e-stat.go.jp/stat-search/files?page=1&layout=datalist&toukei=00400001&tstat=000001011528&cycle=0&tclass1=000001135783&tclass2=000001139046&tclass3=000001139047&tclass4=000001139048&tclass5val=0. Accessed 22 Dec 2021.

[38] Ishikura T. 2,500,000 yen annual aid to 1,000 doctoral students in advanced fields. 2020. https://www.asahi.com/articles/ASNDJ3CQLNDHULBJ00T.html. Accessed 22 Dec 2021.

[39] MEXT. Issues regarding the development and securing of research human resources to strengthen Japan's research capabilities. 2018. https://www.mext.go.jp/b_menu/shingi/gijyutu/gijyutu10/toushin/1410398.htm. Accessed 22 Dec 2021.

[40] NHK. 2,900,000 yen annual aid to 7,000 doctoral students. 2020. https://www.nhk.or.jp/politics/articles/lastweek/50416.html. Accessed 22 Dec 2021.

[41] MEXT. Contribution of doctoral students to research. https://www.mext.go.jp/content/1423020_013.pdf. Accessed 22 Dec 2021.

[42] Tsfati Y, Boomgaarden HG, Strömbäck J, Vliegenthart R, Damstra A, Lindgren E (2020). Causes and consequences of mainstream media dissemination of fake news: literature review and synthesis. Ann Int Commun Assoc.

[43] COVID-19 tracker: Osaka reports record-high 666 new cases Saturday, topping Tokyo's 446, the Japan Times. 2021. https://www.japantimes.co.jp/news/2021/04/03/national/japan-coronavirus-april-3/. Accessed 21 Dec 2021.

[44] Ontario reports 666 new cases of COVID-19, 7 more deaths, Toronto Star. 2021. https://www.thestar.com/news/gta/2021/11/14/ontario-reports-666-new-cases-of-covid-19-7-more-deaths.html. Accessed 21 Dec 2021.

[45] India logs 16,326 new COVID cases, 666 deaths in last 24 hours. The Economic Tines. 2021. https://economictimes.indiatimes.com/news/india/india-logs-16326-new-covid-cases-666-deaths-in-last-24-hours/videoshow/87219606.cms?from=mdr. Accessed 21 Dec 2021.

